# Early Glottic Cancer: Role of MRI in the Preoperative Staging

**DOI:** 10.1155/2014/890385

**Published:** 2014-08-14

**Authors:** Eugenia Allegra, Pierpaolo Ferrise, Serena Trapasso, Orazio Trapuzzano, Antonio Barca, Stefania Tamburrini, Aldo Garozzo

**Affiliations:** ^1^Department of Experimental and Clinical Medicine-Otolaryngology Head and Neck Surgery, University of Catanzaro, Viale Europa, Località Germaneto, 88100 Catanzaro, Italy; ^2^Department of Experimental and Clinical Medicine-Radiology, University of Catanzaro, Viale Europa, Località Germaneto, 88100 Catanzaro, Italy; ^3^Department of Radiology, Pellegrini Hospital, 80135 Naples, Italy

## Abstract

*Introduction.* Clinical staging is the most important time in management of glottic cancer in early stage (I-II). We have conducted a prospective study to evaluate if magnetic resonance imaging (MRI) is more accurate than computed tomography (CT) about tumoral extension, to exactly choose the most appropriate surgical approach, from organ preservation surgery to demolitive surgery. *Materials and Methods.* This prospective study was conducted on 26 male patients, with suspected laryngeal neoplasia of glottic region. The images of MRI and CT were analyzed to define the expansion of glottic lesion to anterior commissure, laryngeal cartilages, subglottic and/or supraglottic site, and paraglottic space. The results of MRI and CT were compared with each other and with the pathology report. *Results.* CT accuracy was 70% with low sensitivity but with high specific value. MRI showed a diagnostic accuracy in 80% of cases, with a sensitivity of 100% and high specificity. Statistical analysis showed that MRI has higher correlation than CT with the pathology report. *Conclusion.* Our study showed that MRI is more sensitive than CT in the preoperative staging of early glottic cancer, to select exactly the eligible patients in conservative surgery of the larynx, as supracricoid laryngectomy and cordectomy by CO_2_ laser.

## 1. Introduction

Laryngeal cancer represents 4.5% of all malignancies and 28% of cancers of the upper aerodigestive tract. Ninety percent of the malignant tumors of the larynx are composed of squamous cell carcinomas, with different distributions of prevalence based on the specific subsite affected (glottic, supraglottic, and subglottic site) [1]. The clinical staging with the assistance of diagnostic imaging is the most important time of therapeutic planning, which should ensure oncological radicality in respect of the clinical outcomes for patients. For this reason, it is necessary to stage the laryngeal cancer in a correct way in order to choose the most correct therapeutic approach based on the available options, from organ preservation strategies (radiotherapy, partial resection/cordectomy with CO_2_ laser, and conservative partial reconstructive surgery) to demolitive surgery. This is especially true for glottic tumors at an early stage of disease, which have demonstrated high rates of local control with organ preservation techniques such as radiotherapy (RT) (84%–95%) and partial resections (85%–100%) [2–4]. Indirect laryngoscopy is the first step in diagnosis and clinical evaluation of the tumor extension, but it is an external investigation and therefore has limitations in the assessment of the implication of the deep structures (such as anterior commissure, thyroid cartilage, and paraglottic spaces), which is discriminating for the extension of surgical resection. A valuable aid is provided by computed tomography (MDCT) and magnetic resonance imaging (MRI) for the evaluation of deep structures, because the involvement of these areas is generally considered as a contraindication for radiotherapy and surgical conservative procedures. It is not a formal contraindication, as supracricoid laryngectomies are still preservation surgery. In these cases, frontolateral vertical laryngectomy or vertical hemilaryngectomy or partial supracricoid laryngectomy with cricohyoidoepiglottopexy (CHEP) or cricohyoidopexy (CHP) is oncologically more suitable, because of removing thyroid cartilage, vocal folds, and paraglottic spaces, although with greater morbidity and increased time of hospitalization. In addition, the reconstruction of the vocal folds allows maintenance of physiological larynx functions like phonation and swallowing, with improvement of the quality of life in these patients [4–7]. For these reasons, it is important to evaluate precisely the extent of tumor preoperatively to plan the correct procedure to assure clear margins to the patient to avoid local recurrence.

CT and MR imaging are routinely used to differentiate between limited and gross cartilage invasion.

Some studies have shown that MRI is more sensitive than CT in the evaluation of cartilage tumor invasion [8–10]. However, cartilage invasion is sometimes overestimated [11–13].

The overestimation of the magnetic resonance protocol is probably related to the presence of peritumoral inflammation, which amplifies/inflates the boundaries of abnormal tissues [14].

We have conducted a prospective study to evaluate if MRI is able to provide more accurate information than CT about the tumoral extension to the anterior commissure, the cartilages, and the possible infiltration of paraglottic spaces in laryngeal glottic cancer at an early stage (I-II); the purpose is to exactly select patients who are eligible for laryngeal conservative surgery (supracricoid laryngectomy and cordectomy by CO_2_ laser), to ensure the oncological radicality and improve clinical outcome.

## 2. Materials and Methods

### 2.1. Patients

The study was conducted at the Department of Otolaryngology, University of Catanzaro (Italy). From August 2011 to November 2013, 26 male patients, aged 52–79 years (median, 63.6 years), with suspected laryngeal cancer of glottic region assessed by indirect laryngoscopy were enrolled; the symptomatology was predominantly characterized by hoarseness and cough. The study was performed with the approval of Institutional Review Board of “Magna Graecia” University of Catanzaro, Italy; all patients give their informed consent to the study.

All patients were subjected to a diagnostic workup including indirect laryngoscopy, MRI and CT of the neck (with and without contrast), and biopsy. In order not to invalidate the results, MRI and CT scans were performed before laryngeal biopsy, so that the images do not prove altered by the presence of peritumoral inflammation. The evaluation of CT and MRI was performed independently by two radiologists who were unaware of the laryngoscopic features and surgical findings. Of 26 patients six were excluded from the study because they were treated with radiotherapy after biopsy confirmed tumor diagnosis (four patients refused surgery and two patients had poor general conditions). Of 20 patients, 14 were smokers, four ex-smokers, and two had never smoked.

Stage of disease in all patients was clinically assessed according to the 7th edition of the TNM classification established by the American Joint Committee on Cancer (AJCC) [15]. The physical examination by indirect laryngoscopy showed unilateral involvement vocal fold in 8/20 (40%) patients and bilateral vocal fold involvement in 12/20 (60%) patients. The T staging by indirect laryngoscopy classified eight patients as T1a, 6 T1b, and 6 T2, with impaired cordal motility.

Patients are currently included in a follow-up program including visits every 3 months with video-laryngoscopy and radiological examinations such as ultrasound of the neck, chest radiography, CT, and MRI, in agreement with clinical evidence.

### 2.2. Staging by MRI

MR images were obtained with a Philips Achieva 1.5 T MR system. MR examinations were performed with an anterior surface neck coil and T1-weighted spin echo and T2 turbo spin echo images in axial and coronal projection, without contrast, diffusion weighted imaging (DWI) and T1w spin echo sequences with fat saturation after paramagnetic contrast infusion of gadolinium chelate were obtained. The number of the sections was 20 for all sequences. The sections were 3-4 mm of interspace thickness with a 1 mm intersection gap. The evaluation of cartilage invasion followed the new criteria proposed by Becker et al. Specifically, T2-weighted or T1-weighted post-Mdc cartilage signal intensity greater than that of the adjacent tumor was considered to indicate inflammation, and signal intensity similar to that of the adjacent tumor was considered to indicate neoplastic invasion [16]. The DWI was performed to better discriminate peritumoral edema from neoplastic tissue, but, at present, there are no studies reporting the performance of DWI. The advantage introduced by DWI sequence consists in obtaining information about the cellularity of tissues [14–16].

The T staging by MRI classified eight patients as T1a, 6 T1b, and 6 T3.

### 2.3. Staging by CT

CT images were obtained with a Toshiba Aquilion CX 64 Multislice CT system. The axial cuts of neck and chest were performed with 2-3 mm of thickness and with 1 mm of intersection gap, before and after intravenous administration of contrast medium [17]. CT criteria used for determining neoplastic invasion of the thyroid cartilage include sclerosis, erosion, lysis, and transmural extralaryngeal tumor spread [17]. The T staging by CT classified 12 patients as T1a and 4 T3; the presence of the disease was not detected in four cases.

### 2.4. Statistical Analysis

The images of MRI and CT were studied to define the expansion of glottic lesion, involvement of anterior commissure, infiltration of laryngeal cartilages and the possible extension to subglottic and/or supraglottic, and the invasion of paraglottic space. The results of MRI and CT were compared with each other and with the definitive pathological examination, each of the two methods for calculating the sensitivity, and the specificity and positive predictive value. For statistical analysis we employed the MedCalc software (version 13.0.6) using the “comparison of proportion” test; the values lower than 0.05 (*P* = 0.05) were considered statistically significant.

## 3. Results

Through histopathological examination, 18 of 20 (90%) were histologically diagnosed as squamous cell carcinoma of the glottis and were staged according to AJCC pTNM staging system, 7th edition [15], resulting in eight as pT1a, 4 pT1b, and 6 pT3; two of the ten patients clinicoradiologically classified as T1a had histopathological diagnosis of squamous cell papilloma.

According to preoperative clinicoradiological staging the classification was T1a in 10 patients (50%), T1b in 4 patients (20%), and T3 in 6 patients (30%). Based on clinicoradiological staging, patients were subjected to excisional biopsy with CO_2_ laser in two cases (classified as squamous cell papillomas by histopathological examination), cordectomy with CO_2_ laser in four cases (4 T1a without involvement of anterior commissure), supracricoid laryngectomy with CHEP and reconstruction of vocal cords in 10 cases (4 T1a, 4 T1b, and 2 T3), and total laryngectomy in four cases (4 T3).

### 3.1. Concordance between MRI and Pathological Staging

MRI classified in a correct way 16 of 20 patients (80%), with four overstaged patients: two lesions classified as cT1b by MR were pT1a and two lesions classified as cT1a were squamous cell papillomas at pathological examination (no tumor).

About the examination of anterior commissure, laryngeal cartilages, and paraglottic space, MRI has been shown to be a very sensitive method (100%), with two false positives, high specificity (97%), and a positive predictive value of 90%, as calculated from the data in Table 1.

### 3.2. Concordance between CT and Pathological Staging

**Figure 1:**
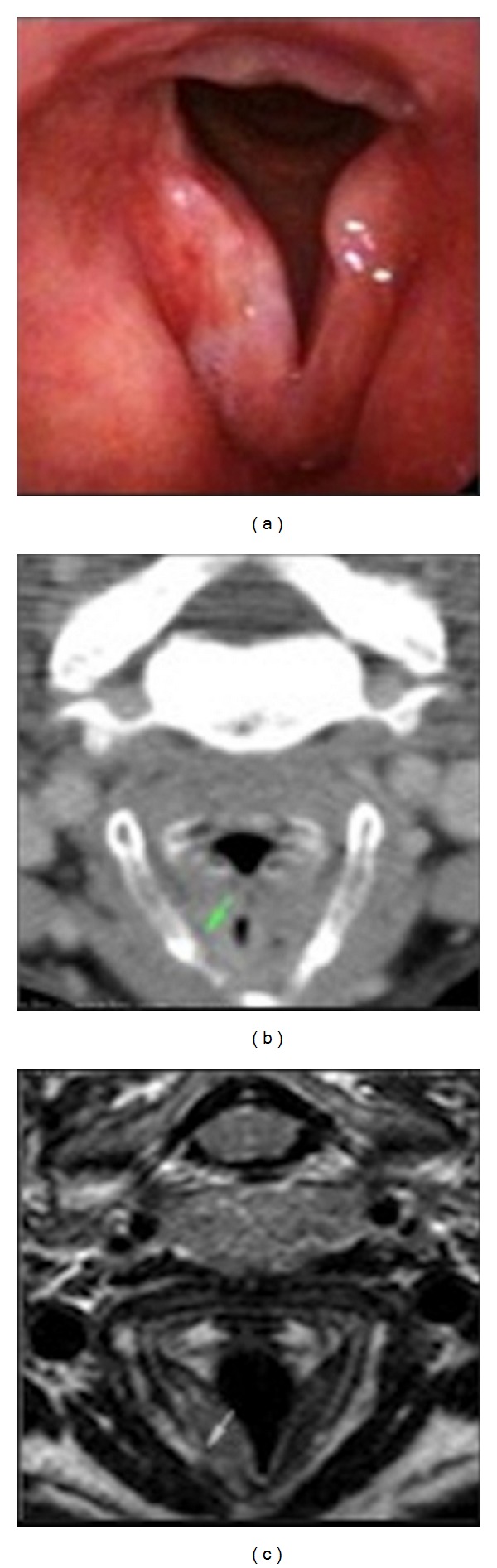
Carcinoma of the right vocal cord: (a) endoscopic view; (b) CT image: the paraglottic space seems preserved (green indicator) without cartilaginous alterations; (c) T2w MR image: the paraglottic space seems involved with focal invasion of the thyroid cartilage (green indicator).

**Figure 2:**
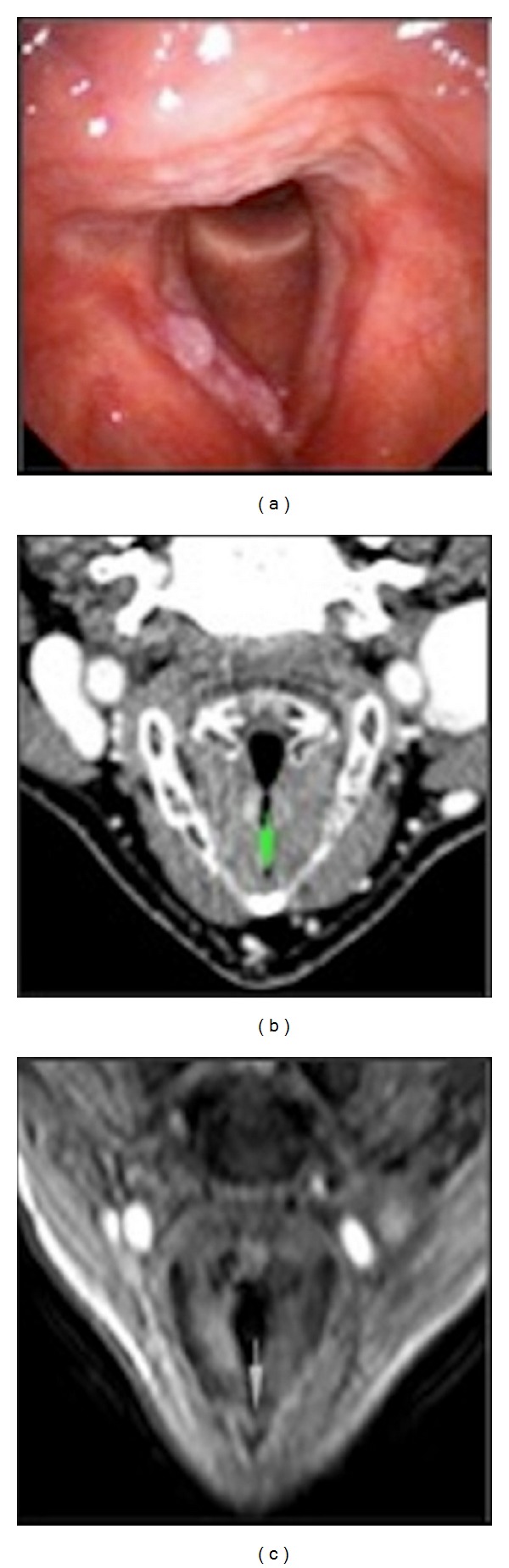
Bilateral glottic carcinoma with involvement of anterior commissure: (a) endoscopic view; (b) CT image: contrast enhancement of right vocal fold, but the commissure seems preserved (green indicator); (c) T1w MR image after contrast shows involvement of right true vocal cord, anterior commissure, and anterior part of left vocal fold (green indicator).

CT classified in a correct way 14 of 20 patients (70%), with six understaged patients: two lesions classified as cT1a by CT were pT1b, two lesions classified as cT1a were pT3, and two tumors were not detected by CT examination. The two cases of squamous cell papillomas were interpreted in a correct way. About the examination of anterior commissure, laryngeal cartilages and paraglottic space, CT has been shown to be a little sensitive method (40%), with eight false negatives (Figures 1 and 2) but with high specificity (100%), as calculated from the data in Table 2.

### 3.3. Data Analysis

In our series, there is a statistically significant difference between MRI and CT in identifying the involvement of anterior commissure (*P* = 0.0098), and a considerable difference also exists in the study of paraglottic space, even if it does not reach statistical significance (*P* = 0.06) (Table 3). Moreover, taking into account the correspondence of the clinical-radiologic T staging with pT staging, a percentage of understadiations equal to 0% for MRI and 33% for CT emerge, which reaches statistical significance (*P* = 0.02). CT scans are charged with a large number of false negatives, while MR has found two cases of false positives. In our study, CT does not overstage the cases in contrast to MRI, but the difference does not reach statistical significance. CT scans showed no lesions in patients with papillomas, while MR showed asymmetry of the glottis with gradient contrast enhancement of the lesion and suspicion of malignancy. CT has estimated the involvement of paraglottic spaces in two cases of six and the infiltration of thyroid cartilage in two cases of six, while MRI is not responsible for errors in these assessments. In two cases there was an involvement of arytenoid cartilage; both CT and MRI have been equaled in these assessments. Results of data comparison are summarized in Table 4.

## 4. Discussion

This prospective study evaluates the contribution by MRI and CT in the clinical staging of early glottic cancer (T1-T2) for the evaluation of submucosal areas that can change the stage of the disease and reassess the therapeutic approach. In particular, MRI shows a sensitivity of 100% and a specificity of 97% in assessing areas such as paraglottic space, anterior commissure, thyroid, and arytenoid cartilages, with various indications for conservative surgery. Instead, the sensitivity of CT reaches lower values, 40%, but it has high specificity (100%). In our series, CT staging was accurate in 70% of cases, while the MRI was accurate in 80% of cases. By Kuno et al., the accuracy of CT in staging was 80% and 87.5% for the MR, without significant differences between RMI and TC in the assessment of anterior commissure and paraglottic space, while for the determination of cartilaginous invasion MR showed a higher sensitivity than CT, which instead resulted to be significantly more specific. However cartilage invasion is sometimes overestimated, resulting in unnecessary total laryngectomies in some patients [13].

Again, the integration of DWI into the magnetic resonance protocol has the potential to increase the specificity [16].

The ability of CT in the evaluation of the cartilage invasion has been studied by several authors resulting in a variable sensitivity from 46% to 74% and a specificity variable from 87% to 94% [8, 10, 18–20]. By applying Becker's criteria for cartilage invasion for the evaluation of all laryngeal cartilages (extralaryngeal spreads and erosion/lysis) and for the evaluation of cricoid and arytenoid (single sclerosis), the sensitivity arrives at 82% and specificity at 79% [17]. Hartl et al. evaluated the role of CT for detecting cartilage invasion in early glottic tumors and showed a sensitivity of 10.5% and a specificity of 94%, overestimating the cartilage invasion in case of injury involving the commissure and overestimating it in case of lesion with impaired vocal fold mobility; they emphasized the inability of CT in the evaluation of focal invasion of the internal perichondrium in thyroid cartilage [21].

About RMI, Castelijns et al. asserted that CT and MRI were equally specific, while MRI was more sensitive than CT [8]. Becker claimed higher sensitivity of MRI than CT (89% versus 66%) but less specificity (84% versus 94%) [10]. In 1998, Declercq et al. showed a sensitivity of MRI equal to 100% [22] and in 2001 Atula et al. showed equivalence of the sensitivity and specificity of 67% [23]. Banko et al. have demonstrated accuracy equal to 100% in the evaluation of anterior commissure using MRI [24], similar to that found by Zbaren et al. equal to 83% [18]. About the evaluation of early glottic carcinomas, Bertrand et al. considered CT as a first line investigation, using only later on MRI for the examination of dubious areas such as the anterior commissure, subglottis, and arytenoid cartilages [20]. Anterior commissure is an area of particular interest, because its particular conformation may be a resistant space to tumoral deep space extension, until the thyroid cartilage, and configures it as a decisive factor for the choice of surgical resection. In fact, X-space's dense fibrous structures act as a barrier to extension in depth and may lead to spreading along the surface on the mid line till subglottic region [25–27]. This behavior contraindicates conservative surgery. In our series, CT has understaged the invasion of thyroid cartilage and paraglottic space; in a patient with bilateral glottic cancer, CT has not evaluated the invasion of anterior commissure, and in another case of glottic tumor it has not identified any tumoral alterations.

In the literature, the debate about the best therapeutic approach for glottic cancer that involves the anterior commissure is still open. In a review, 64 patients with T1 glottic cancer were treated with radiotherapy; 14 patients had involvement of commissure and they had a local control rate of 76% at 2 years and 58% at 5 years, with statistically significant difference [28]. In another review, 53 patients with glottic carcinoma were treated with radiotherapy: 8/14 (57.1%) patients with involvement of anterior commissure had locoregional recurrence of disease [29]. In another series, 200 cases classified as T1 were treated with radiotherapy and they had a local control rate of 89% in case of commissure involvement compared to 94%, when the commissure was not involved [30]. The anterior commissure, therefore, remains as one of the most adverse independent prognostic factors [31]. The CO_2_ laser treatment for glottic carcinomas involving the anterior commissure is supported by Steiner et al., while Eckel argues that it is burdened by high rates of recurrence (37.1%) [32, 33]. Sachse et al. compare the effectiveness of two conservative treatments, CO_2_ laser excision and partial laryngectomy, in 119 tumors classified as T1-T2, and they show that the involvement of anterior commissure greatly reduces the local control rate in patients treated with CO_2_ laser resection [34]. Zohar et al. and Laccourreye et al. reported a local control rate of 90% with supracricoid laryngectomy (SCL) in case of glottic tumors involving the anterior commissure, compared to 72% with radiotherapy [35, 36]. The modified supracricoid laryngectomy (MSCL) with reconstruction of vocal folds results oncologically safe in case of involvement of anterior commissure without recurrence at five years and with a local control rate equal to 90.5%, also increasing the quality of voice and life than the SCL [4–7].

Based on these considerations, it is clear in the treatment of early glottic cancer that it is important to identify the involvement of anterior commissure, paraglottic spaces, and laryngeal cartilages: the possible involvement of these deep structures could contraindicate CO_2_ laser treatment or radiation, because of its high rate of recurrence or chondronecrosis. Any focal involvement of arytenoid cartilages or paraglottic space and thyroid cartilage requires a more radical treatment, using LSC or MSCL, preserving functional laryngeal functions [5].

## 5. Conclusion 

In our study, statistical analyses have showed that MRI could be considered a helpful diagnostic method for preoperative staging of laryngeal tumor and decision making of the best therapeutic option in these patients. MR imaging has several advantages with respect to multislice CT and few limitations (artifacts related to movement, namely, breathing, swallowing, and vessel pulsation). MRI allows a multiparameter analysis (T1 weighted, T2 weighted, DWI, and postcontrast acquisition). This multiparameter approach amplifies the contrast resolution. Moreover the lack of accurate evaluation radiologic imaging and substaging of disease can change therapeutic planning and patients survival. Data from our study are very encouraging. Even though MRI is more expensive, longer, and not always feasible for patients compared to CT scan (poor compliance, any contraindications), based on the above considerations, we believe that it could be considered the investigation of choice in the clinical evaluation of early glottic lesions for the planning of therapeutic interventions, because of its high sensitivity and elevated degree of diagnostic accuracy. However, the evaluation of MR images needs high experience and an interdisciplinary collaboration.

## Figures and Tables

**Table 1:** Concordance between MRI and pathological staging: true positive (TP), false positive (FP), true negative (TN), and false negative (FN), according to laryngeal subsites.

Site	TP	FP	TN	FN
	Number	Number	Number	Number
Paraglottic space	6	0	14	0
Thyroid cartilage	4	0	16	0
Arytenoid cartilage	2	0	18	0
Cricoid cartilage	0	0	20	0
Anterior commissure	8	2	10	0

**Table 2:** Concordance between CT and pathological staging: true positive (TP), false positive (FP), true negative (TN), and false negative (FN), according to laryngeal subsites.

Site	TP	FP	TN	FN
	Number	Number	Number	Number
Paraglottic space	2	0	14	4
Thyroid cartilage	2	0	16	2
Arytenoid cartilage	2	0	18	0
Cricoid cartilage	0	0	20	0
Anterior commissure	2	0	12	6

**Table 3:** Concordance between pathological, MRI, and CT staging with *P* value, according to laryngeal subsites.

Laryngeal site	Pathological involvement	MRI	CT	*P* value
	Number	Number (%)∗	Number (%)∗	
Paraglottic space	6	6 (100%)	2 (33%)	0.06
Thyroid cartilage	4	4 (100%)	2 (50%)	0.41
Arytenoid cartilage	2	2 (100%)	2 (100%)	n.v.
Cricoid cartilage	0	0 (100%)	0 (100%)	n.v.
Anterior commissure	8	8 (100%)	2 (25%)	**0.0098**

(%)∗: accuracy.

**Table 4:** Percentage of concordance between pathological, MRI, and CT T staging with *P* value.

T staging	CT	MR	*P* value
Correct stadiations	88%	66%	0.24
Understadiations	11%	0%	0.47
Overstadiations	0%	33%	**0.02**

## References

[1] Ortholan C, Benezery K, Dassonville O (2011). A specific approach for elderly patients with head and neck cancer. *Anticancer Drugs*.

[2] Hartl DM, Ferlito A, Brasnu DM (2011). Evidence-based review of treatment options for patients with glottic cancer. *Head & Neck*.

[3] Chone CT, Yonehara E, Martins JEF, Altemani A, Crespo AN (2007). Importance of anterior commissure in recurrence of early glottic cancer after laser endoscopic resection. *Archives of Otolaryngology—Head and Neck Surgery*.

[4] Allegra E, Franco T, Trapasso S, Domanico R, La Boria A, Garozzo A (2012). Modified supracricoid laryngectomy: oncological and functional outcomes in the elderly. *Clinical Interventions in Aging*.

[5] Garozzo A, Allegra E, La Boria A, Lombardo N (2010). Modified supracricoid laryngectomy. *Otolaryngology—Head and Neck Surgery*.

[6] Allegra E, Lombardo N, La Boria A (2011). Quality of voice evaluation in patients treated by supracricoid laryngectomy and modified supracricoid laryngectomy. *Otolaryngology—Head and Neck Surgery*.

[7] Allegra E, Franco T, Trapasso S, Aragona T, Domanico R, Garozzo A (2012). Quality of life in patients treated by organ preservation surgery for early laryngeal carcinoma. *Open Access Surgery*.

[8] Castelijns JA, Gerritsen GJ, Kaiser MC (1988). Invasion of laryngeal cartilage by cancer: comparison of CT and MR imaging. *Radiology*.

[9] Zbären P, Becker M, Läng H (1997). Staging of laryngeal cancer: endoscopy, computed tomography and magnetic resonance versus histopathology. *European Archives of Oto-Rhino-Laryngology*.

[10] Becker M, Zbaren P, Laeng H, Stoupis C, Porcellini B, Vock P (1995). Neoplastic invasion of the laryngeal cartilage: comparison of MR imaging and CT with histopathologic correlation. *Radiology*.

[11] Li B, Bobinski M, Gandour-Edwards R, Farwell DG, Chen AM (2011). Overstaging of cartilage invasion by multidetector CT scan for laryngeal cancer and its potential effect on the use of organ preservation with chemoradiation. *British Journal of Radiology*.

[12] Hartl DM, Landry G, Hans S, Marandas P, Brasnu DF (2010). Organ preservation surgery for laryngeal squamous cell carcinoma: low incidence of thyroid cartilage invasion. *Laryngoscope*.

[13] Kuno H, Onaya H, Fujii S (2013). Primary staging of laryngeal and hypopharyngeal cancer: CT, MR imaging and dual energy CT. *European Journal of Radiology*.

[14] Maroldi R, Ravanelli M, Farina D (2014). Magnetic resonance for laryngeal cancer. *Current Opinion in Otolaryngology & Head and Neck Surgery*.

[15] Sobin LH, Gospodarowicz MK, Wittekind C (2009). *TNM Classification of Malignant Tumours*.

[16] Becker M, Zbären P, Casselman JW, Kohler R, Dulguerov P, Becker CD (2008). Neoplastic invasion of laryngeal cartilage: reassessment of criteria for diagnosis at MR imaging. *Radiology*.

[17] Becker M, Zbären P, Delavelle J (1997). Neoplastic invasion of the laryngeal cartilage: reassessment of criteria for diagnosis at CT. *Radiology*.

[18] Zbaren P, Becker M, Lang H (1996). Pretherapeutic staging of laryngeal carcinoma clinical findings,
computed tomography, and magnetic resonance imaging compared with
histopathology. *Cancer*.

[19] Sulfaro S, Barzan L, Querin F (1989). T-staging of the laryngohypopharyngeal carcinoma. A 7-year multidisciplinary experience. *Archives of Otolaryngology—Head and Neck Surgery*.

[20] Bertrand M, Tollard E, François A (2010). CT scan, MR imaging and anatomopathologic correlation in the glottic carcinoma T1-T2. *Revue de Laryngologie Otologie Rhinologie*.

[21] Hartl DM, Landry G, Bidault F (2013). CT-scan prediction of thyroid cartilage invasion for early laryngeal squamous cell carcinoma. *European Archives of Oto-Rhino-Laryngology*.

[22] Declercq A, van den Hauwe L, van Marck E, van de Heyning PH, Spanoghe M, de Schepper AM (1998). Patterns of framework invasion in patients with laryngeal cancer: correlation of in vitro magnetic resonance imaging and pathological findings. *Acta Oto-Laryngologica*.

[23] Atula T, Markkola A, Leivo I, Mäkitie A (2001). Cartilage invasion of laryngeal cancer detected by magnetic resonance imaging. *European Archives of Oto-Rhino-Laryngology*.

[24] Banko B, Đukić V, Milovanović J, Kovač JD, Artiko V, Maksimović R (2011). Diagnostic significance of magnetic resonance imaging in preoperative evaluation of patients with laryngeal tumors. *European Archives of Oto-Rhino-Laryngology*.

[25] Bridger GP, Nassar VH (1972). Cancer spread in the larynx. *Archives of Otolaryngology*.

[26] Bagatella F, Bignardi L (1983). Behavior of cancer at the anterior commissure of the larynx. *Laryngoscope*.

[27] Bagatella F, Bignardi L (1981). Morphological study of the laryngeal anterior commissure with regard to the spread of cancer. *Acta Oto-Laryngologica*.

[28] Nozaki M, Furuta M, Murakami Y (2000). Radiation therapy for T1 glottic cancer: involvement of the anterior commissure. *Anticancer Research*.

[29] Maheshwar AA, Gaffney CC (2001). Radiotherapy for T1 glottic carcinoma: impact of anterior commissure involvement. *Journal of Laryngology and Otology*.

[30] Gowda RV, Henk JM, Mais KL, Sykes AJ, Swindell R, Slevin NJ (2003). Three weeks radiotherapy for T1 glottic cancer: the Christie and Royal Marsden Hospital experience. *Radiotherapy and Oncology*.

[31] Bradley PJ, Rinaldo A, Suárez C (2006). Primary treatment of the anterior vocal commissure squamous carcinoma. *European Archives of Oto-Rhino-Laryngology*.

[32] Steiner W, Ambrosch P, Rödel RMW, Kron M (2004). Impact of anterior commissure involvement on local control of early glottic carcinoma treated by laser microresection. *Laryngoscope*.

[33] Eckel HE (2001). Local recurrences following transoral laser surgery for early glottic carcinoma: frequency, management, and outcome. *Annals of Otology, Rhinology and Laryngology*.

[34] Sachse F, Stoll W, Rudack C (2009). Evaluation of treatment results with regard to initial anterior commissure involvement in early glottic carcinoma treated by external partial surgery or transoral laser microresection. *Head and Neck*.

[35] Zohar Y, Rahima M, Shvili Y, Talmi YP, Lurie H (1992). The controversial treatment of anterior commissure carcinoma of the larynx. *Laryngoscope*.

[36] Laccourreye O, Muscatello L, Laccourreye L, Naudo P, Brasnu D, Weinstein G (1997). Supracricoid partial laryngectomy with cricohyoidoepiglottopexy for “early” glottic carcinoma classified as T1-T2N0 invading the anterior commissure. *The American Journal of Otolaryngology—Head and Neck Medicine and Surgery*.

